# Special Issue “Drug Discovery and Application of New Technologies”

**DOI:** 10.3390/ijms252111756

**Published:** 2024-11-01

**Authors:** Sha Hu, Yaxin Li, Liming Hu

**Affiliations:** Beijing Key Laboratory of Environmental and Viral Oncology, College of Chemistry and Life Science, Beijing University of Technology, Beijing 100124, Chinayaxin_li@emails.bjut.edu.cn (Y.L.)

## 1. Overview of Technological Innovations in Drug Discovery

Historically, drug discovery and development have proven to be time-consuming and costly, with the process averaging around 15 years and costing approximately USD 2 billion to bring a new small-molecule drug to market [1]. The landscape of drug discovery has undergone a significant transformation in recent years, largely driven by advances in computational approaches [2] and structural biology techniques. These innovations are reshaping the traditional approaches, enabling more efficient and targeted drug development. The most impactful advancement is the comprehensive application of artificial intelligence technology in new drug research and development.

Artificial Intelligence for Drug Discovery (AIDD) leverages the collection and organization of extensive biochemical datasets, compound representation, and advanced AI algorithms, including generative adversarial networks and other deep learning models, to generate novel compound structures. Additionally, it develops machine learning models that assist scientists in predicting drug–target structures, drug–target interactions, binding affinities, drug toxicity, drug bioactivity, and physicochemical properties [3,4]. Proteomics and metabolomics employ technologies such as mass spectrometry and nuclear magnetic resonance to investigate small molecules and proteins within biological samples, significantly advancing the drug discovery process by elucidating targets, drug mechanisms, and biomarkers [5,6]. Next-generation sequencing technology facilitates the rapid acquisition of genomic, transcriptomic, and epigenomic information, yielding a vast array of data for target identification. This technology also enables scientists to elucidate drug resistance mechanisms, optimize drug combinations, and enhance therapeutic efficacy, thereby expediting the development of new drugs [7,8,9]. In the field of nanotechnology, nano-carriers such as nanoparticles, nano-emulsions, liposomes, and micelles significantly enhance the stability and solubility of drug molecules, thereby improving their bioavailability and playing a crucial role in drug discovery [10,11,12].

As the integration of artificial intelligence and structural biology techniques continues to advance, the drug discovery process becomes more streamlined. Trends like the use of ultra-large virtual screening libraries and the application of deep learning for protein structure prediction are driving the field forward. These innovations promise to accelerate the discovery of new drugs and broaden the scope of therapeutic targets, including those considered challenging or “undruggable”.

## 2. Key Challenges and Future Directions

Despite significant progress in the field of drug discovery, several challenges continue to hinder the seamless translation of these innovations into clinical success. (1) Artificial Intelligence for Drug Discovery remains heavily reliant on high-quality chemical and biological data, and the current scarcity of biological data and limited samples for machine learning pose significant challenges to its further development. (2) In genomics and transcriptomics, the substantial volume of data generated by high-throughput sequencing necessitates proficiency in bioinformatic tools for effective data processing and analysis. Individual variability and biological variation can result in inconsistent outcomes, thereby affecting data authenticity and the feasibility of clinical applications. In metabolomics and proteomics, scientists encounter challenges in sample handling and data analysis, which demand a high level of expertise in research when applying omics technologies to drug discovery. (3) The intricate design and modification of biological systems in synthetic biology necessitate the effective integration of multiple biological components and pathways. Addressing the challenges of translating laboratory findings in synthetic biology into clinical applications and drug development will be crucial areas of research in the future. (4) The synthesis of nanomaterials presents challenges due to its relative complexity and high cost, and the long-term biocompatibility and toxicity of these materials within biological systems remain inadequately understood. Moreover, effectively controlling the timing and rate of drug release continues to be a significant challenge.

## 3. Overview of the Contributions in the Issue

This Special Issue brings together groundbreaking studies that explore novel methodologies, technologies, and compounds in drug discovery, with applications ranging from viral infection treatment to cancer therapy. The advancements presented here reflect the power of integrating cutting-edge technologies, including molecular docking, ultrafiltration techniques, and bispecific antibody–drug conjugates (bsADCs), in regard to handling some of the most pressing challenges in therapeutic development.

A study by Wang et al. demonstrates the power of traditional compounds in modern drug discovery. Curcumin, derived from turmeric, was shown to inhibit Porcine Deltacoronavirus (PDCoV) replication through a combination of molecular docking and network pharmacology techniques. This study identifies curcumin’s binding to key viral targets, such as IL-6 and TNF signaling pathways, providing an avenue for antiviral drug development [13]. Zhuang et al. present a novel approach to improving the efficacy of antibody–drug conjugates (ADCs) for HER2-low-expressing tumors. By engineering a SORT1×HER2 bispecific antibody–drug conjugate, they demonstrate enhanced internalization and antitumor activity compared to conventional HER2-targeted therapies. This technology represents a promising strategy for treating HER2-low-expressing cancers that are resistant to standard therapies [14]. Alostath et al. found that CHX-CaCl_2_ surface crystallization is a new drug technology for controlled and sustained CHX release; its antibacterial effectiveness makes the drug an ideal adjunct following clinical and surgical procedures in terms of maintaining oral hygiene and preventing surgical site infections [15]. Lu et al. conducted an initial exploration of potential α-glucosidase inhibitors stemmed from siraitia grosvenorii roots. Sixteen potential inhibitors were successfully isolated from siraitia grosvenorii roots, including lignans, cucurbitacins, and cucurbitane glycosides [16]. Li et al.’ study provides insights into the dimerization patterns of chemokine receptors and the functional significance of their truncated isoforms [17]. Bai et al. demonstrated that an amyloid antibody could be engineered by a few mutations to bind new amyloid sequences, providing an efficient way to reposition a therapeutic antibody to target different amyloid diseases [18]. Liu et al. developed a formulation of liposomes co-loaded with Panax notoginseng saponins (PNSs) and a Ginsenoside Rg3 (Lip-Rg3/PNS) novel nano-delivery system to treat ischemic stroke via intranasal administration [19]. Finally, Torres-Jaramillo et al. reported the synthesis of 2-(4-alkyloxyphenyl)-imidazoline and imidazole derivates and found two new and potent antiprotozoal on *L. mexicana* and *T. cruzi* [20].

## 4. Final Reflections

As we look to the future, drug discovery and the application of new technologies will enhance our understanding of the potential of drug discovery in meeting the growing demand for safe and effective therapeutics against human diseases. The integration of new technologies into drug discovery holds the promise of bringing more effective and personalized treatments to patients faster than ever before. We hope that the collection of articles will serve as a valuable resource for researchers, fostering innovation and collaboration in the pursuit of better therapies.
